# 

**Published:** 1908-10-17

**Authors:** 


### BOOKS RECEIVED.

W. Heinemann.

"My Story." By Hall Caine.

Henry Kimpton.

"Neurasthenia." By Gilbert Ballet. Translated from the French by P. Campbell-Smith, M.D.

T. Nelson and Sons.
(Nelson Shilling Library.)

"Scrambles Amongst the Alps." By Edward Whymper.
"Collections and Recollections." By G. W. E. Russell.
"The Great Boer War." By Sir Arthur Conan Doyle.
"The Life of John Nicholson." By Captain Trotter.

### THE GRADUATES' MEDICAL SCHOOLS.

DIARY FOR WEEK OCT. 19 TO OCT. 24.

### THE WEST-END HOSPITAL FOR NERVOUS DISEASES, Welbeck Street, W.

**At 5 p.m.**

**Oct. 22,** Dr. T. D. Savill, **Concerning Cases Illustrating Symptoms of Diseases of the Ends of the Extremities.**

### NATIONAL HOSPITAL FOR THE PARALYSED AND EPILEPTIC, Queen Sq., Bloomsbury, W.C.

**At 3.30 p.m.**

**Oct. 20 and 23,** Dr. Beevor, **Cerebral Anatomy and Localisation.**

### THE POST-GRADUATE COLLEGE, West London Hospital, Hammersmith, W.

**At 12 noon.**

**Oct. 19,** Dr. Low, **Pathological Demonstration.**

**At 12.15 p.m.**

**Oct. 21 and 23,** Dr. Pritchard, **Practical Medicine.**

**At 5 p.m.**

**Oct. 19,** Mr. Lloyd, **Anæsthetics—I.**

**Oct. 20,** Mr. Armour, **Whitlow.**

**Oct. 21,** Mr. Williams, **When to Extract.**

**Oct. 22,** Mr. Baldwin, **Practical Surgery.**

**Oct. 23,** Mr. Armour, **Gangrene.**

### THE THROAT HOSPITAL, Golden Square, W.

**At 5.30 p.m.**

**Oct. 19,** Mr. Faulder, **Anatomy and Physiology of the Nose.**

**Oct. 22,** Mr. Parker, **Methods of Eaamination.**

### NORTH-EAST LONDON POST-GRADUATE COLLEGE, Prince of Wales's General Hospital, Tottenham, N.

**At 4.30 p.m.**

**Oct. 20,** Mr. A. de Prenderville, **The Anæsthetic Technique for Operations on the Throat and Nose.**

**Oct. 22,** Dr. Giles, **Preparation for Abdominal Operations.**

### ST. JOHN'S HOSPITAL FOR DISEASES OF THE SKIN, Leicester Square, W.C.

**At 6 p.m.**

**Oct. 22,** Dr. Dockrell, **Eczema—its Varieties, Symptoms, and Causes.**

### MEDICAL GRADUATES' COLLEGE AND POLYCLINIC, 22 Chenies Street, W.C.

**At 5.15 p.m.**

**Oct. 19,** Dr. R. Hutchison, **Achylia.**

**Oct. 20,** Dr. G. H. Savage, **Characteristics of Toxic Insanities.**

**Oct. 21,** Dr. T. G. Stevens, **The Causes and Treatment of Abortion.**

**Oct. 22,** Dr. Chas. Mercier, **Moral Imbecility.**

### LONDON SCHOOL OF CLINICAL MEDICINE, Seamen's Hospital, Greenwich, S.E.

**At 2.15 p.m.**

**Oct. 21,** Dr. F. Taylor, **The Bladder in Medical Cases.**

**At 3.15 p.m.**

**Oct. 23,** Mr. McGavin, **The Surgery of Rectal Stenosis.**

---

THE HOSPITAL October 17, 1908.

*Name* ..........................................................

*Address* ..........................................................

..........................................................

This Coupon must accompany manuscript or contributions intended for The Hospital.

